# Selected peptide-based fluorescent probes for biological applications

**DOI:** 10.3762/bjoc.16.247

**Published:** 2020-12-03

**Authors:** Debabrata Maity

**Affiliations:** 1 Department of Chemistry, New York University, New York, NY 10003, USA

**Keywords:** fluorescent probe, fluorophores, molecular recognition, peptide-based

## Abstract

To understand the molecular interactions, present in living organisms and their environments, chemists are trying to create novel chemical tools. In this regard, peptide-based fluorescence techniques have attracted immense interest. Synthetic peptide-based fluorescent probes are advantageous over protein-based sensors, since they are synthetically accessible, more stable, and can be easily modified in a site-specific manner for selective biological applications. Peptide receptors labeled with environmentally sensitive/FRET fluorophores have allowed direct detection/monitoring of biomolecules in aqueous media and in live cells. In this review, key peptide-based approaches for different biological applications are presented.

## Introduction

Molecular recognition involving amino acids or peptides are important factors in biochemical and medicinal processes [1–3]. Amino acids work as biosynthetic building blocks or as signaling molecules. For example, the well-known neurotransmitters glutamate and γ-aminobutyric acid associate with membrane-bound protein receptors and trigger changes in receptor shape and activity with subsequent signaling across the membrane. Noncovalent H-bonding and van der Waals interactions are the basis for the selective molecular recognition between a G-coupled protein receptor and a bound glutamate molecule [4]. Peptides often work as signaling molecules or hormones, such as small neuropetide endorphins, produced by the central nervous system to relieve stress or enhance pleasure. They produce signaling cascades in the brain by interacting with opiate receptors. Sometimes membrane-bound receptors interact strongly with short peptidic segments of a larger protein chain, for example, recognition of the RGD sequence (arginine–glycine–aspartic acid; Arg–Gly–Asp) by integrin receptors. They use noncovalent interactions including salt bridges [5]. Vancomycin, a glycopeptide antibiotic, is used for the treatment of resistant bacterial infections. Its interaction with a small peptidic segment of the bacteria cell wall is a classic example of molecular recognition [6–7]. Peptides are often substrates for protease enzymes [8–9]. Enzymologists have studied the chemical principles behind the substrate recognition and catalytic reactivity for a long time. Hence, there is a wealth of knowledge on the type of interaction of small peptide sequences with specific partners. Researchers designed several artificial peptide-based receptors for molecular recognition purposes [10–13]. Peptides are comparatively small and can be easily synthesized in the laboratory using standard synthetic protocols [14–15]. The peptide sequence can be manipulated with precise functional groups of amino acids for selective targeting biomolecules. Peptides are large enough to contain a significant number of precisely located functional groups of amino acid sidechains that can codify high-affinity and specific interactions with a target receptor. There are plenty X-ray or NMR structural information available and can be exploited for the rational design of specific peptidic receptors. Integrating an organic fluorophore (reporter) with a specific peptide sequence is the common approach for designing peptide-based fluorescent probes [16]. Fluorophores on peptidic arms perform as reporter during the interaction with biomolecules [17–18]. The interaction with biomolecules will alter the fluorescence intensity, lifetime or excitation/emission maxima. The fluorescence turn-on response offers a bright signal against a dark background, which maximizes spatial resolution than the fluorescence turn-off signal [19]. A shift in the absorption/emission spectrum is also advantageous as the changes in ratio of intensities of absorption or emission at two wavelengths minimizes the error from the physical or chemical fluctuations in the sample.

**Figure 1:**
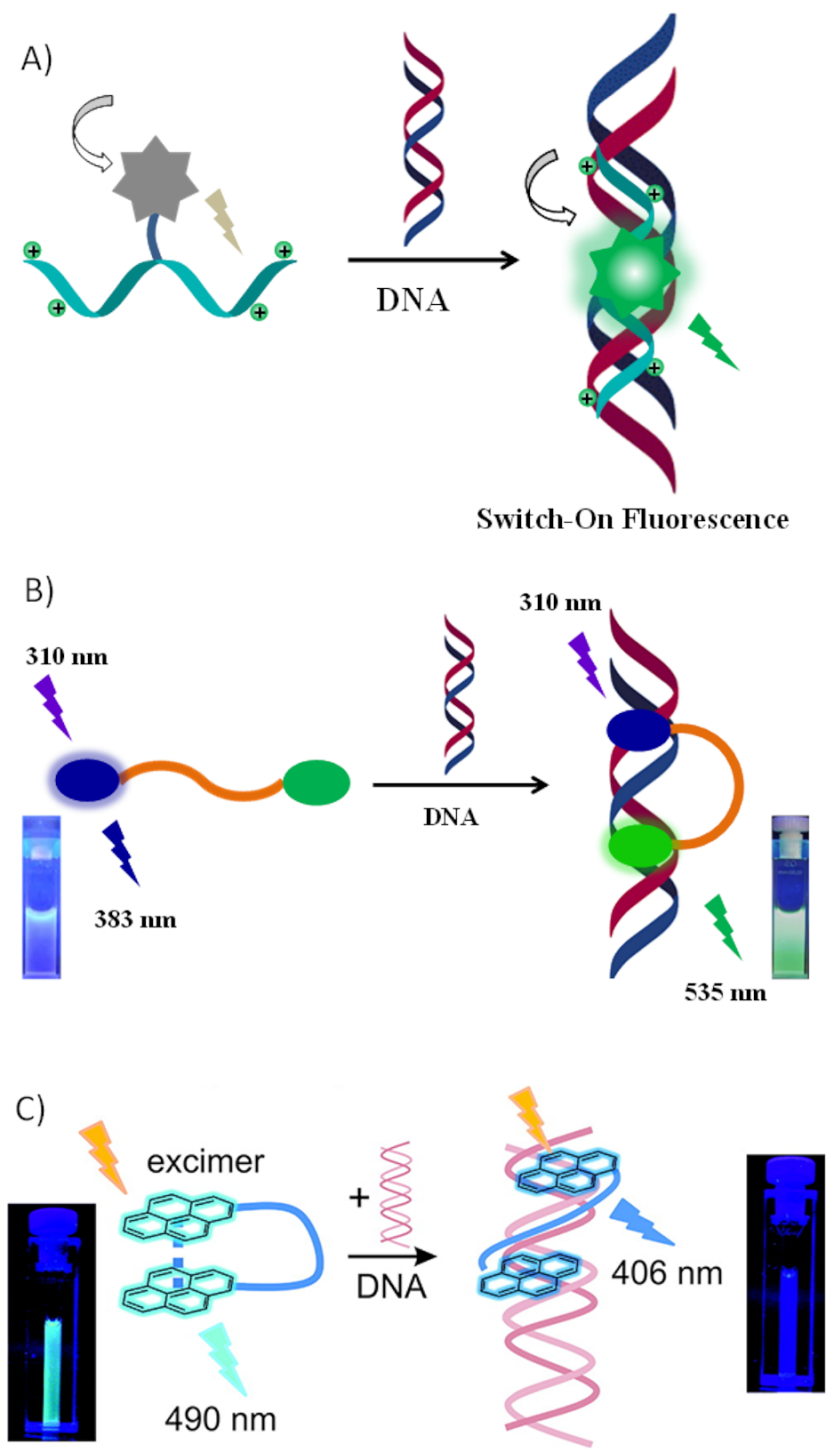
Three different type of peptide-based fluorescent probes and their interaction with nucleic acids are shown. A) Environment-sensitive fluorophore attached peptide shows “switch-on” fluorescence response upon interaction with nucleic acids. Reproduced with permission from [32], Maity et al., “Peptide‐Based Probes with an Artificial Anion‐Binding Motif for Direct Fluorescence “Switch‐On” Detection of Nucleic Acid in Cells”, Chem. – Eur. J. © 2017 Wiley‐VCH Verlag GmbH & Co. KGaA, Weinheim; B) FRET pair attached peptide shows ratiometric fluorescence response upon interaction with nucleic acids. Reproduced from [33] with permission from The Royal Society of Chemistry; C) a pyrene-attached peptides shows ratiometric fluorescence response upon interaction with nucleic acids. Reprinted with permission from [34]. Copyright (2012) American Chemical Society.

Conventional peptide probes based on environment-sensitive fluorophores [20–22], fluorescence resonance energy transfer (FRET) pairs [13] and pyrene excimer/monomer [23] formation have been frequently adopted. Several classes of environment-sensitive fluorophores are currently available and heavily used by the biological community. Upon interaction with a biomolecule, the fluorophore induces a change in the spectral properties. The fluorescence intensity is generally enhanced and blue-shifts after affinity/recognition in a polar solvent. Several computational studies confirmed that the spectral change is caused by the insertion of the environment-sensitive fluorophore in the hydrophobic domains/grooves of the target protein/DNA (Figure 1A). The major problem of these fluorophores: they interact noncovalently, exposure of labeled cells to solvents may also extract these fluorophores. However, peptidic attachment of these fluorophores has greatly mitigated loss of fluorophores [24–26]. Peptide-based environment-sensitive fluorescent probes have been successfully used for biomolecule detection [27–28]. Being intrinsically modular, peptide scaffolds also allow the attachment of multiple fluorophores simultaneously [29]. It provides the opportunity of developing ratiometric fluorescent sensors from the interaction between multiple fluorophores (Figure 1B). Fluorescence resonance energy transfer (FRET) relies on the distance-dependent transfer of energy from a donor fluorophore to an acceptor fluorophore. Genetically encoded fluorophores, such as green fluorescent protein (GFP) and related blue, cyan, yellow and red fluorescent proteins have provided the ability to perform FRET in vitro and in vivo, particularly in living cells [30]. FRET-based peptide probes are heavily used for ratiometric fluorescence detection of biomolecules. Pyrene-functionalized oligonucleotides and locked nucleic acids (LNAs) are considerably used for targeting nucleic acids [31]. The attachment of pyrenes to the termini of a flexible peptide linker results in a ratiometric fluorescent probe (Figure 1C). Upon interaction with a biomolecule, the distance between the pyrenes changes, subsequently their monomer to excimer and vice versa fluorescence signal changes. However, the use of pyrene as a fluorophore has two major disadvantages such as poor quantum yield and an emission in the blue region, which is unfavorable for potential applications in biological systems. The Schmuck group successfully developed several peptide-based probes using these approaches for targeting important biomolecules including nucleotides, DNA, proteins, lipids etc. in aqueous media. Fluorescent peptides have also been used for specific organelles such as lysosomes tracking. In this review, it is summarized Schmuck group’s tremendous effort in developing fluorescent peptide-based probes for biological applications.

## Review

### Nucleoside triphosphates detection

**Figure 2:**
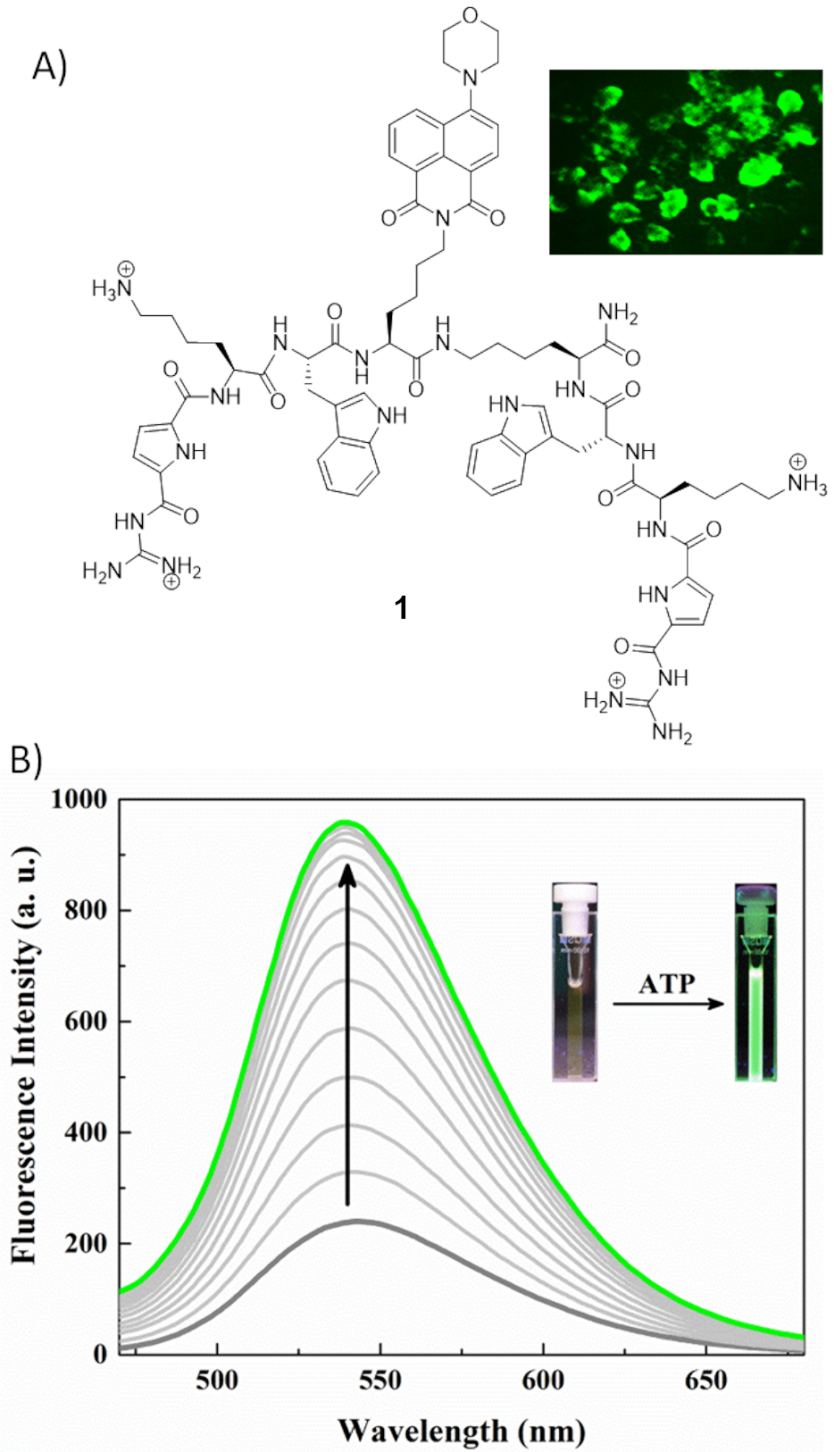
A) Molecular structure of peptidic probe **1**, Inset: HeLa cells incubated with peptide **1** (50 μM), showing an ATP responsive green fluorescence; B) fluorescence emission spectra of probe **1** (20.0 µM) (λ_ex_ = 410 nm) with increasing concentration (0–10.0 µM) of ATP in 10 mM HEPES buffer, pH 7.4. Reproduced from [39] with permission from The Royal Society of Chemistry.

Nucleoside triphosphates play crucial roles in several biological processes including energy transduction, cellular respiration, enzyme catalysis, and signaling [35–38]. They are the most targeted anionic species because of their ubiquitous presence in biological systems. Schmuck and co-workers reported a tweezer type peptide-based probe **1** for fluorescence detection of nucleoside triphosphates in aqueous media (Figure 2) [39]. The probe **1** consists of an environment-sensitive amino-naphthalimide fluorophore having two symmetric peptidic arms equipped with lysine and an artificial strong anion binding site, the guanidinocarbonylpyrrole (GCP) moiety (Figure 2A). These arms also contain tryptophan for dissimilar aromatic interactions with different nucleobases. The fluorescence intensity of probe **1** increases by more than 4-fold at 540 nm in presence of 10 µM ATP (Figure 2B). The ATP binding constant is calculated to be 2.2 × 10^5^ M^−1^. A fluorescence job plot between **1** and ATP confirms an 1:1 binding stoichiometry between ATP and probe **1**. The fluorescence response follows the following order ATP > GTP > UTP > CTP > TTP > PPi >> ADP > UDP. Nucleobases undergo differential interactions with the tryptophan residues and naphthimide which varies the hydrophobic microenvironment around the fluorophore and results in dissimilar fluorescence enhancement. The addition of monophosphorylated species such as HPO_4_^2−^, c-AMP, AMP, CMP, GMP, UMP, TBAP, MMP or other anions (SO_4_^2−^, NO_3_^−^, HCO_3_^−^, CH_3_COO^−^) does not show any enhancement of fluorescence enabling the probe as selective for nucleoside triphosphates. The probe **1** is not toxic and has been demonstrated by fluorescence imaging of ATP in HeLa cells (Figure 2A, inset).

### Nucleic acid detection

**Figure 3:**
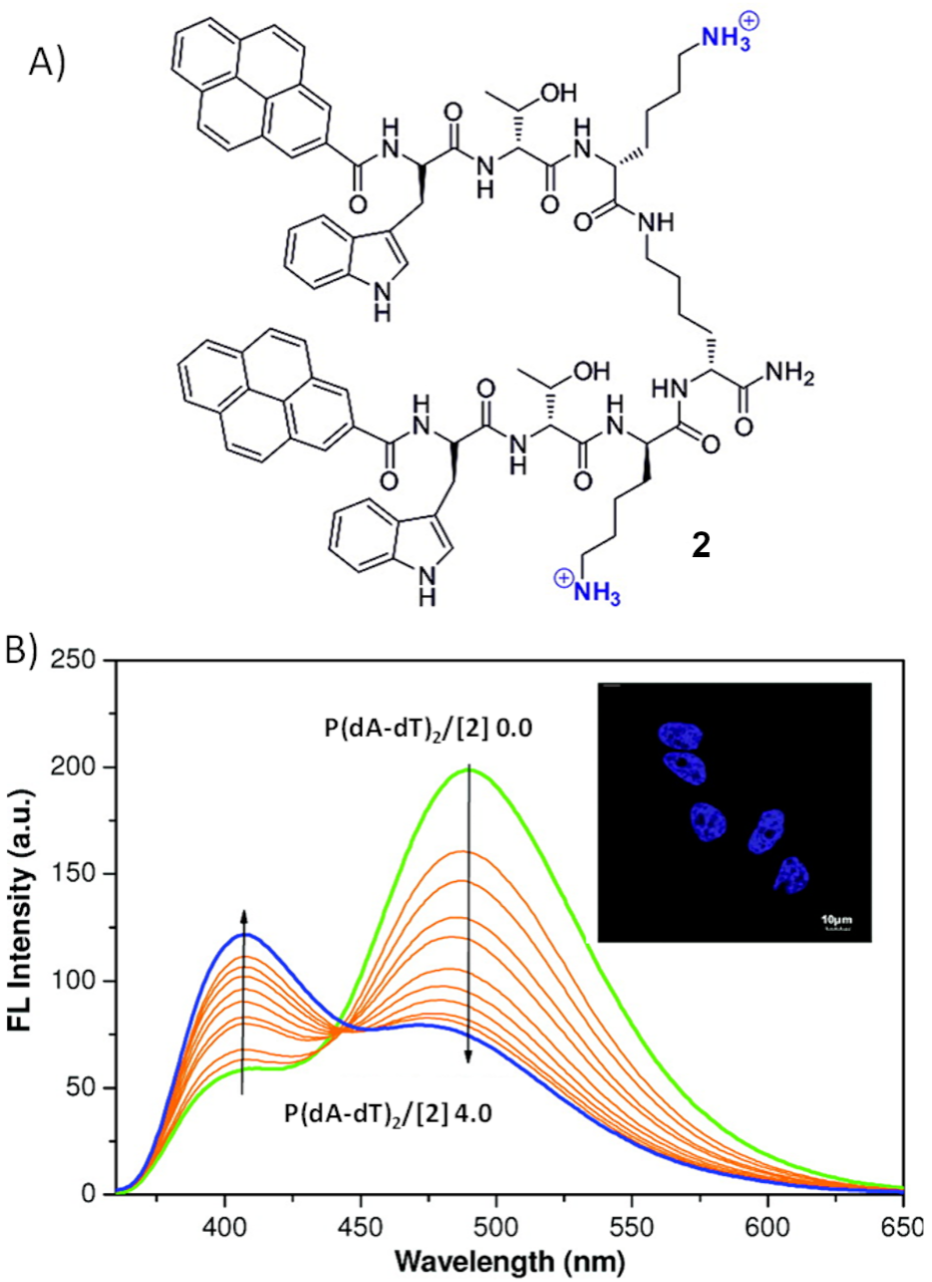
A) Molecular structure of probe **2**; B) fluorescence emission spectra for the titration of a 10 μM solution of **2** with p(dA·dT)_2_ in aqueous buffer (pH 7.4) (with base pair/**2** molar ratios ranging from 0 to 4.0), inset: nuclei of HeLa cells stained with **2**. Reprinted with permission from [34]. Copyright (2012) American Chemical Society.

The genetic information carrier nucleic acids are present in nearly all living organisms. Fluorescence monitoring of nucleic acids has become increasingly important in medical diagnosis, drug discovery, environmental monitoring, food safety etc. [40–41]. Schmuck et al. reported several fluorescent probes for nucleic acids detection. The first probe **2** is a pyrene-based peptide beacon containing two Trp–Thr–Lys tripeptide arms attached to a central lysine spacer (Figure 3A) [34]. Unbound peptide beacon **2** in folded form exhibits typical pyrene excimer emission at 490 nm. Peptide beacon **2** interacts effectively with double-stranded DNA (dsDNA) as positively charged lysine residues are expected to interact with the phosphate backbone electrostatically. Upon binding to dsDNA, it undergoes a conformational change from the folded to an extended form that switches the fluorescence signal from excimer (490 nm) to monomer emission (406 nm, Figure 3B). Thus, monitoring the relative fluorescence intensities at two wavelengths (F_406_/F_490_) allowed the ratiometric detection of nucleic acids.

**Figure 4:**
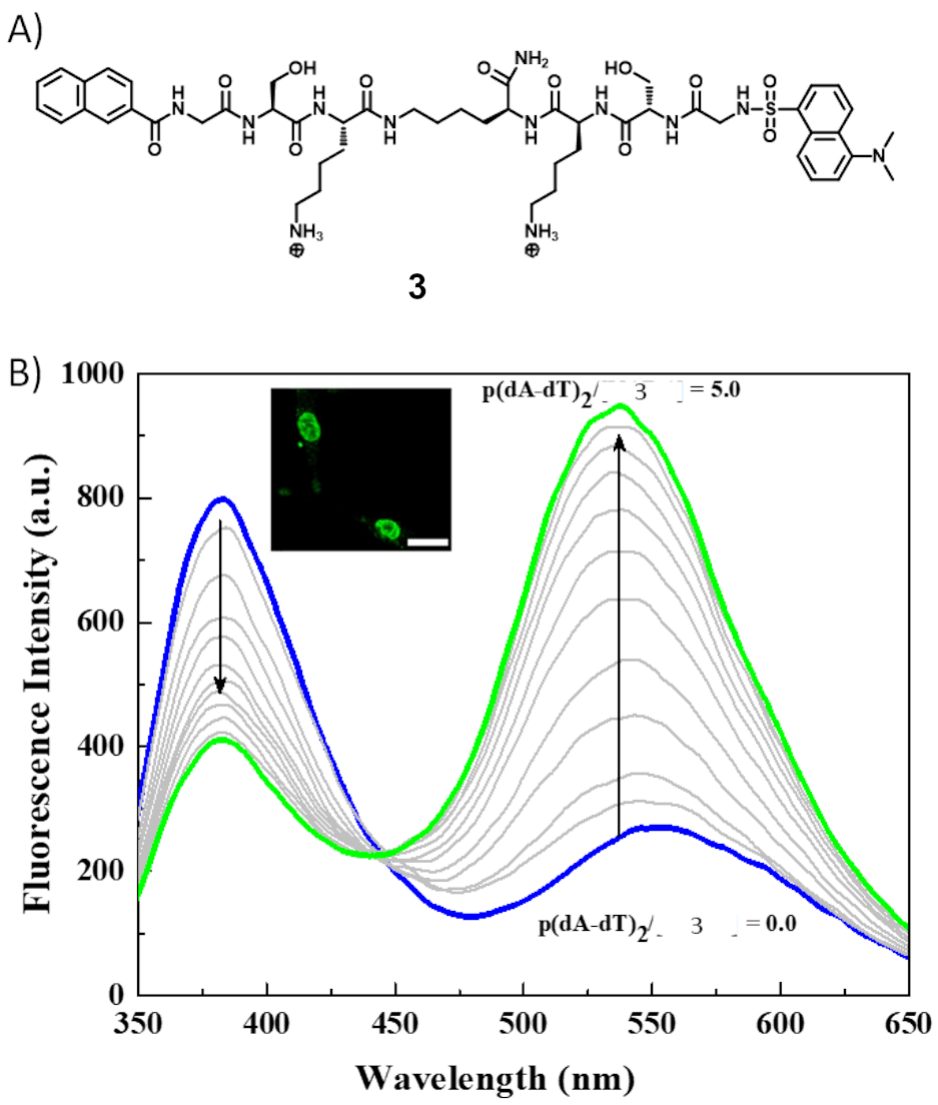
A) Molecular structure of **3**; B) fluorescence emission spectra for the titration of a 10 μM solution of **3** with p(dA·dT)_2_ in 10 mM HEPES buffer (pH 7.4) (with base pair/**3** molar ratios ranging from 0 to 5.0), inset: Nuclei of A549 cells stained with **3**. Reproduced from [33] with permission from The Royal Society of Chemistry.

Schmuck et al. reported a similar cationic peptide beacon **3** coupled with a FRET pair, a naphthalene donor and a dansyl acceptor, for ratiometric detection of dsDNA (Figure 4 [33]. Compound **3** contains two Gly–Ser–Lys tripeptide arms attached via their C-terminus to a central lysine spacer (Figure 4A). Compound **3** is in unfolded form in unbound conditions as it exhibits the naphthalene emission at 383 nm upon 310 nm excitation. However, it folds upon binding to dsDNA, thereby changing the efficiency of the FRET process between the two fluorophores and exhibiting a significant red shift in the emission spectrum (Figure 4B). Compound **3** emits at 535 nm upon 310 nm excitation in the presence of dsDNA. These two peptide beacons (**2** and **3**) based fluorescent probes exhibit a more pronounced change in fluorescence signals for AT-rich polynucleotides than GC-rich polynucleotides. Their fluorimetric responses are proportional to the AT-base pair content in DNA. Both peptides have been demonstrated for staining nuclear DNA (Figure 3B, Figure 4B).

**Figure 5:**
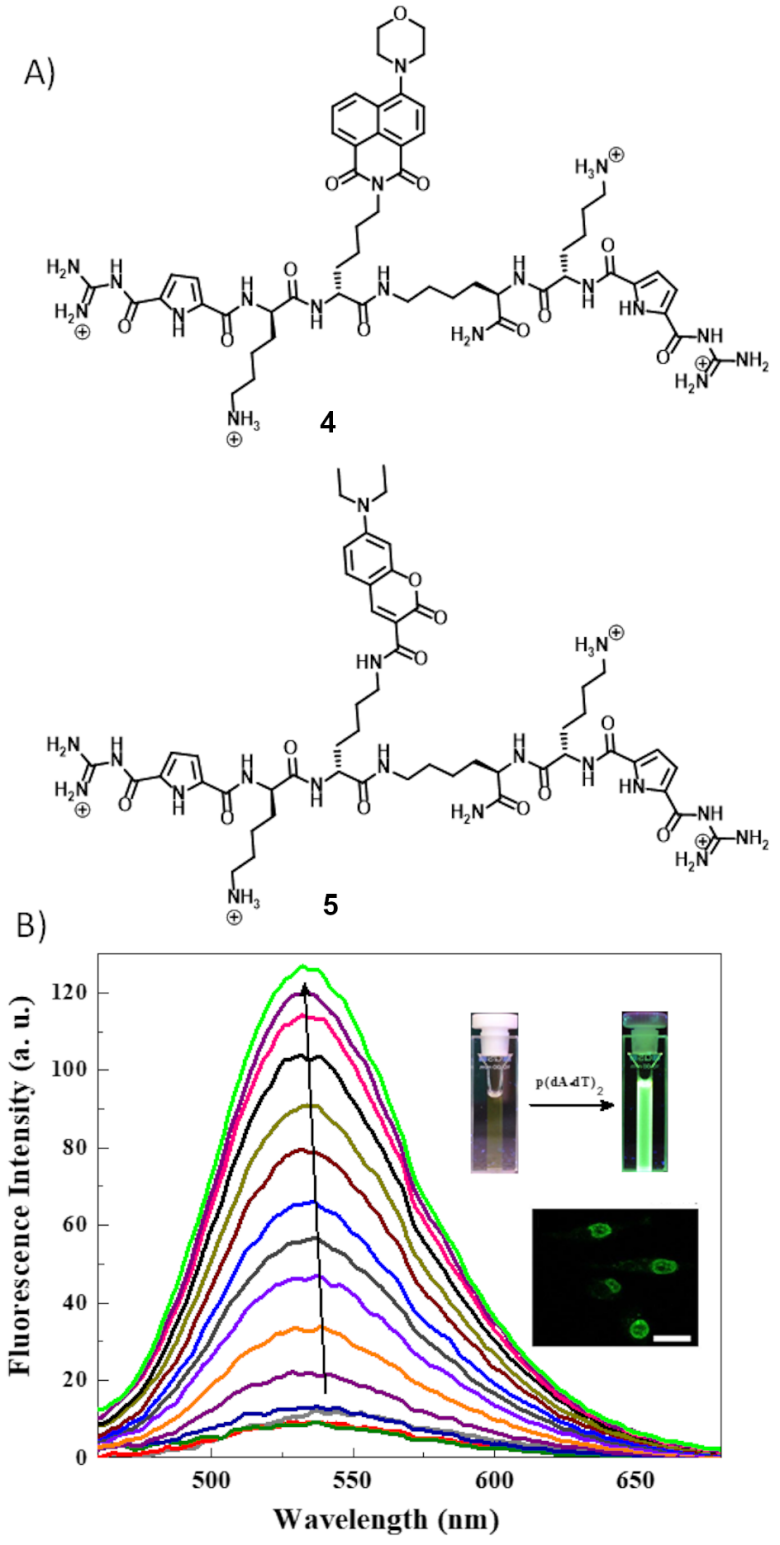
A) Molecular structure of **4** and **5**; B) fluorescence spectra for the titration of a 0.5 μM solution of **4** with increasing concentration of p(d*A*·d*T*)_2_ (λ_ex_ = 410 nm). Inset: fluorescence switched-on after addition of dsDNA inside the cuvette, nuclei of A549 cells stained with **4**. Reproduced with permission from [32], Maity et al., “Peptide‐Based Probes with an Artificial Anion‐Binding Motif for Direct Fluorescence “Switch‐On” Detection of Nucleic Acid in Cells”, Chem. – Eur. J. © 2017 Wiley‐VCH Verlag GmbH & Co. KGaA, Weinheim.

The Schmuck group in collaboration with the Piantanida group developed two tweezer-type fluorescent probes (**4** and **5**) for fluorescent detection of dsDNA (Figure 5) [32]. These two probes have two identical peptide arms each equipped with lysine and an artificial anion binding GCP moiety. In between these two arms, a fluorophore (aminonaphthalimide for **4** and diethylaminocoumarin for **5**) is linked to a third arm. Compounds **4** and **5** are weakly fluorescent and show significant “switch-on” fluorescence response upon interaction with dsDNA (Figure 5B). Probe **4** shows preference for AT-rich polynucleotides (log *K* = 4.6) than GC-rich polynucleotides (log *K* = 3.8). But probe **5** shows comparable binding affinity towards these two polynucleotides (log *K* = 4.7 and log *K* = 4.8, respectively, for p(dA·dT)_2_ and p(dG·dC)_2_). However, they both shows weaker affinity for dsRNA (polyA-polyU) (log *K* = 3.8 and log *K* = 4.4 respectively for **4** and **5**). Significant differential fluorescence responses of these probes with nucleic acids are due to the positioning of fluorophores within the polynucleotide binding site. Different studies explain their different mode of binding with dsDNA. Probe **4** binds with dsDNA in a combined intercalation and minor groove binding mode, whereas probe **5** binds with the outer surface of dsDNA. These two probes are low cytotoxic and probe **4** has been successfully used for imaging of nuclear DNA (Figure 5B, inset).

### Protein detection

#### Specific peptides and insulin

**Figure 6:**
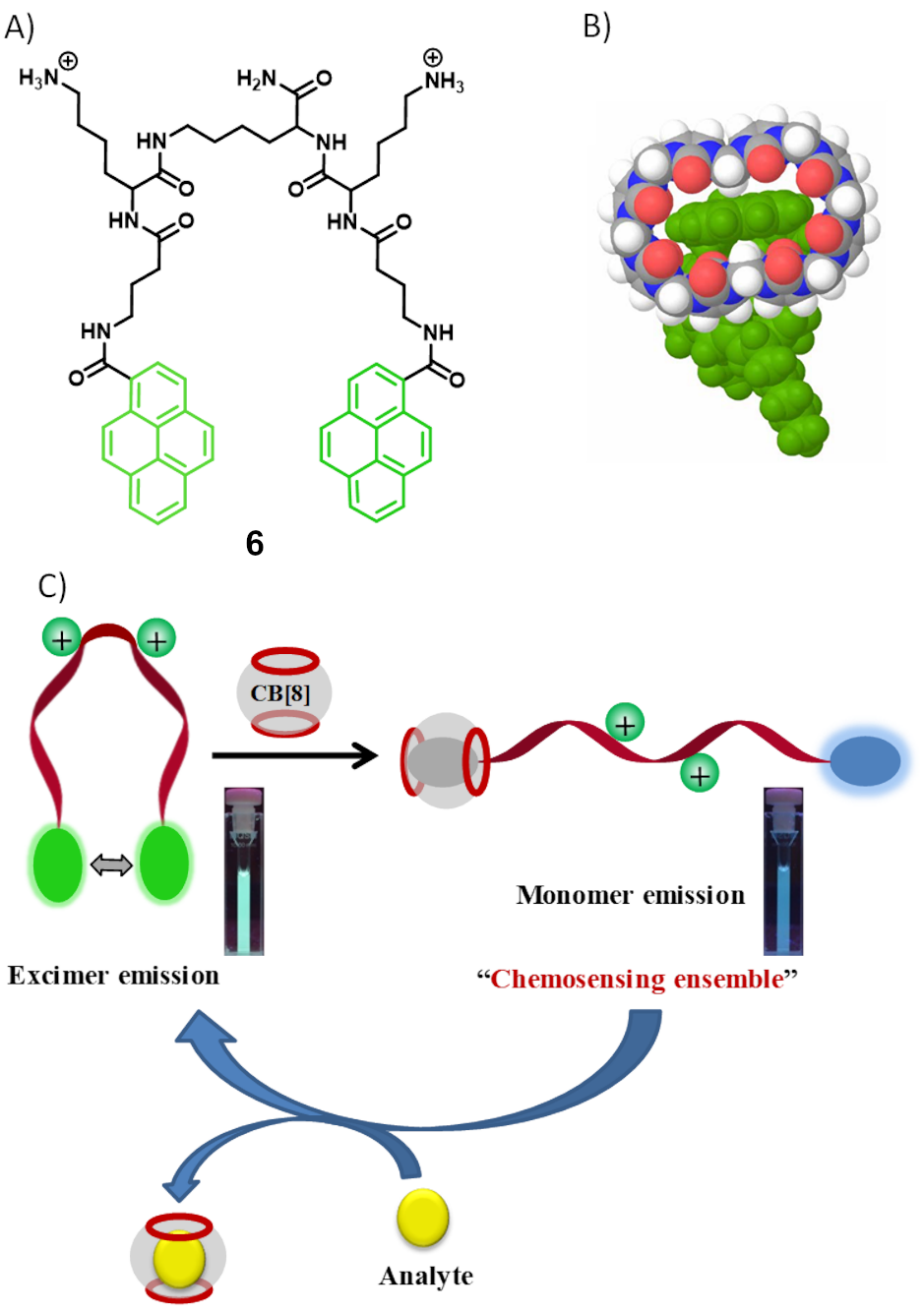
A) Molecular structure of **6**; B) possible binding mode of pyrene termini of **6** to CB[8] according to molecular modeling; C) cartoon representation of ratiometric fluorescent detection application using **6** and the CB[8] conjugate. (The photographs show the corresponding cuvettes under UV light). Reproduced from [42], © 2019 Maity et al., distributed under the terms of the Creative Commons Attribution 4.0 International Licence, http://creativecommons.org/licenses/by/4.0/.

Schmuck and co-workers reported a supramolecular ensemble in combination of a pyrene-tagged amphiphilic peptide beacon (**6**) and a macrocyclic host (cucurbit[8]uril, CB[8]) for ratiometric fluorescent detection of amino acid derivatives, specific peptides, and proteins in aqueous media (Figure 6) [42]. Probe **6** has a central lysine spacer which is connected to two symmetric peptidic arms. Each arm contains a cationic lysine as a positively charged head group and a pyrene moiety at the end of the γ-aminobutyric acid linker. Compound **6** is mostly in folded conformation in solution and emits pyrene excimer fluorescence at 505 nm. Upon addition of CB[8], the fluorescence color changes from pyrene excimer to monomer emission as the hydrophobic CB[8] cavity encapsulates one pyrene terminus. The Schmuck group uses the **6**·CB[8] conjugate for ratiometric fluorescence monitoring of different substrates which have affinity for the CB[8] cavity. These substrates easily displace **6** from the CB[8] cavity and release **6** which folds again to form a pyrene excimer, which allows for the ratiometric fluorescence monitoring of those substrates. Upon screening methyl ester derivatives of hydrophobic amino acids (TrpOMe, PheOMe, LeuOMe) these are found to be more active compared to other amino acid derivatives in displacing **6** from the CB[8] cavity. Similarly, peptides having a hydrophobic amino acid residue at the N-terminus also show a high displacement activity. Human insulin, having a rare Phe residue at the N-terminus of its B-chain, also shows ratiometric fluorescence behavior but in a reverse manner. This kind of ratiometric fluorescence property was not observed for other tested blood proteins (BSA, IgG and BCA).

#### 14-3-3 proteins

**Figure 7:**
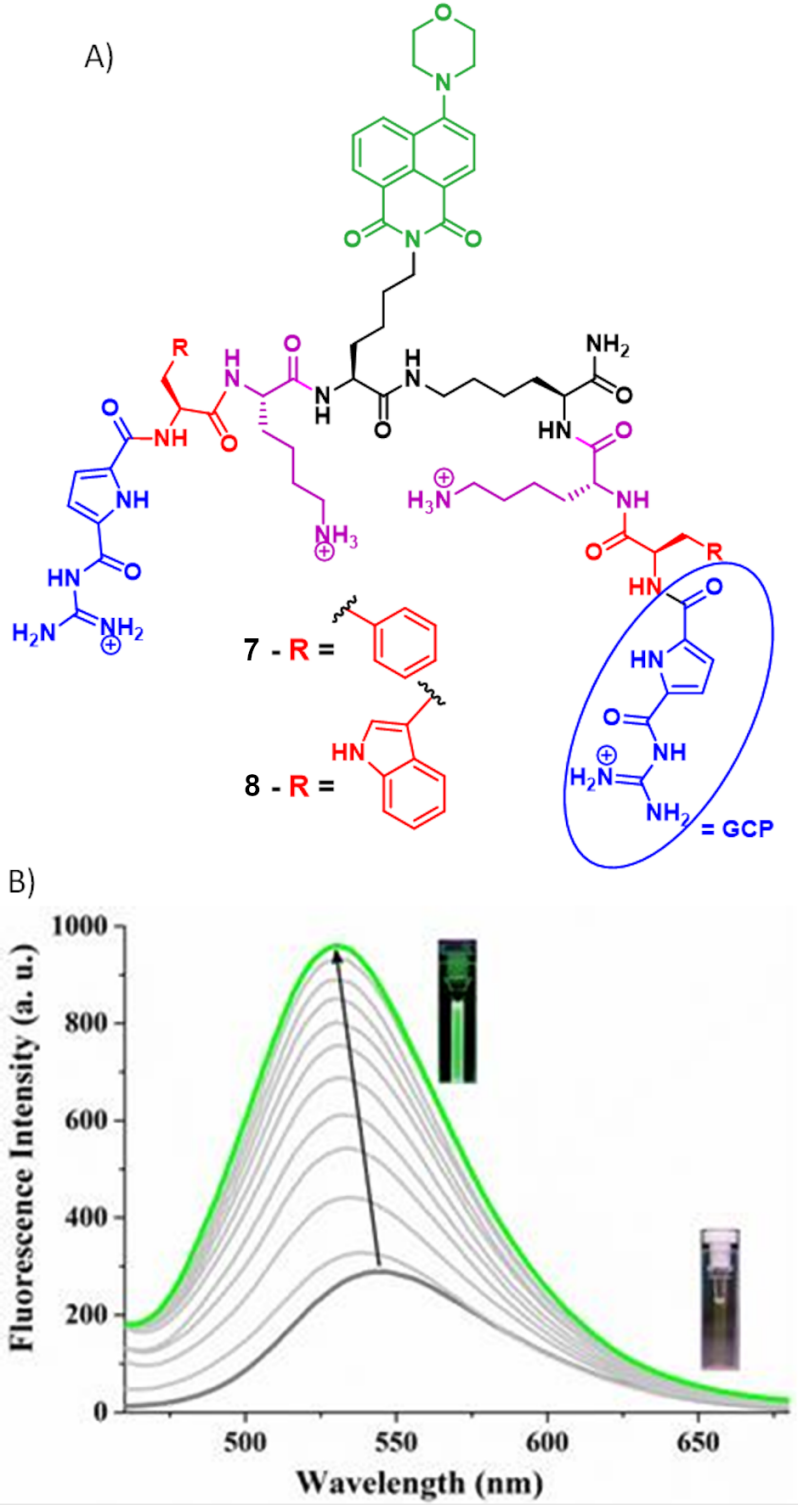
A) Molecular structure of peptidic probes **7** and **8**; B) fluorescence emission spectra of probe **7** (5.0 µM) with increasing concentration (0–5.0 µM) of 14-3-3β protein (λ_ex_ = 410 nm). Inset: Corresponding fluorescence color of the solution inside cuvette under UV lamp. Reproduced from [47] with permission from The Royal Society of Chemistry.

14-3-3 proteins are a family constituted of 7 isoforms (beta, epsilon, gamma, tau, theta, sigma, and zeta) [43–44]. This protein family plays key roles in human physiology including metabolism, stress response, protein trafficking, cell-cycle control, signal transduction, apoptosis, and neurotransmission by binding to their phosphorylated protein partners [45]. They are involved in a variety of human diseases like cancer, Alzheimer’s, and Parkinson diseases etc. [46]. Therefore, the Schmuck group in collaboration with the Ottmann group sought to develop fluorescent probes (**7** and **8**) for monitoring of 14-3-3 proteins (Figure 7) [47]. These probes contain two symmetric GCP-Phe/Trp–Lys–Gly peptidic arms and an artificial anion binding GCP moiety at both termini for 14-3-3 proteins recognition. They have attached an environment sensitive aminonaphthimide fluorophore on a third arm as fluorescence-based reporter. Compounds **7** and **8** show a weak fluorescence band around 545 nm. However, the fluorescence of these probes increases to a different extent upon addition of different 14-3-3 protein isoforms and the emission spectrum is blue-shifted to 530 nm (Figure 7B). Computation studies confirmed that enhancement of the fluorescence is due to close proximity of the naphthimide fluorophore to a hydrophobic binding domain in the protein. Among all isoforms 14-3-3η protein shows maximum fluorescence enhancement for probe **7** (by 4.8-fold) and for probe **8** (by 6.0-fold). These two probes are selective over other proteins including for β-tryptase, trypsin, chymotrypsin, bovine serum albumin. Other biomolecules such as adenosine, adenosine monophosphate, adenosine triphosphate, phosphate, pyrophosphate, glucose, heparin, hyaluronic acid and chondroitin sulfate, and amino acids containing two carboxylates (glutamic acid and aspartic acid) have not increased the fluorescence intensity. Control studies confirmed that the tailor-made GCP moiety is crucial for selective recognition of 14-3-3 proteins. A high cell viability is observed for these two probes when tested with HeLa cell lines in cytotoxicity studies.

#### β-Tryptase

**Figure 8:**
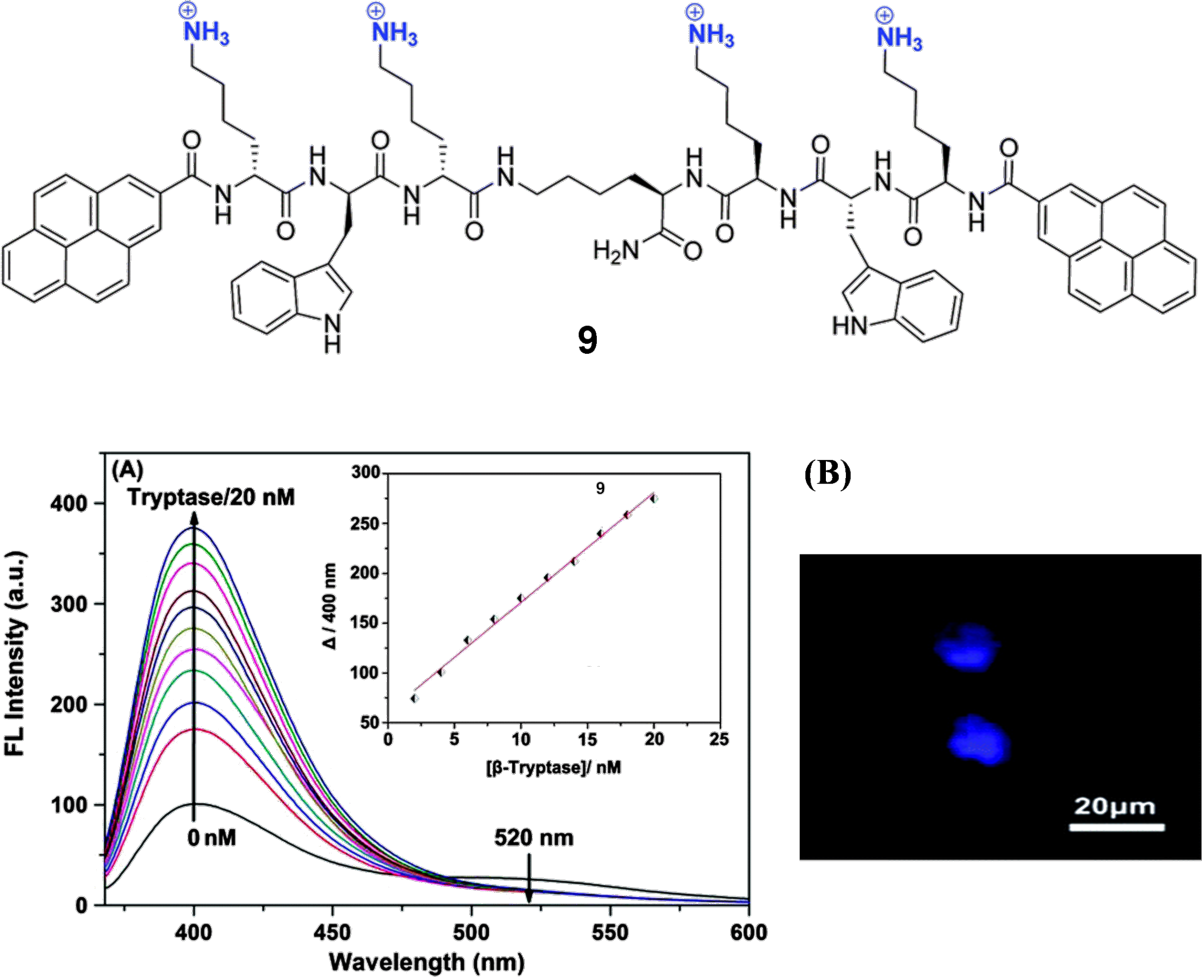
Top: Molecular structure of **9**; bottom: A) fluorescence response of **9** (500 nM) upon addition of β-tryptase (0–20 nM). Inset: the corresponding titration curve showing the increase of the emission at 400 nm with increasing β-tryptase concentration; B) confocal laser scanning microscopy images of CHMAS cells treated with 10 μM peptide **9** for 30 min. Reproduced from [51], “A fluorescent light-up probe as an inhibitor of intracellular β-tryptase”, © 2014 Wang et al., licensed under a Creative Commons Attribution 3.0 Unported Licence, https://creativecommons.org/licenses/by/3.0/. Published by The Royal Society of Chemistry.

β-Tryptase is the predominant secretory granule-derived serine protease of human mast cells and is known to be involved in the pathogenesis of asthma and other allergic and inflammatory disorders [48–50]. The structure of the enzyme is tetramer, composed of four identical subunits arranged in two different orientations, around a central pore. In vivo heparin stabilizes the tetrameric structure, without its tryptase dissociates into inactive monomers. The reported cationic ligand binds at the rim of anionic residues around the entrance of the central pore, blocks the access to the active sites, and inhibits enzyme activity. Schmuck et al. reported a cationic fluorescent peptidic β-tryptase inhibitor (peptide **9**, Figure 8) [51]. The peptide is designed by attaching two Lys–Trp–Lys tripeptide units to a central lysine via the C-terminus. The N-terminus of both sides are functionalized with pyrenes. Peptide **9** exhibits pyrene monomer emission at 400 nm and pyrene excimer weaker emission at 520 nm. In presence of β-tryptase (0–20 nM), the monomer emission increases significantly while excimer emission disappears (Figure 8A). The enhancement of the pyrene monomer fluorescence confirms the involvement of the pyrenes in the β-tryptase binding. The monomer emission increases due to the increased hydrophobic microenvironment around the pyrenes and the restriction of their intramolecular rotation under bound conditions. The structurally similar two proteins trypsin and chymotrypsin as well as bovine serum albumin does not exhibit any significant changes in fluorescence when tested with the peptide. Peptide **9** also inhibits rhSkin β-tryptase enzymatic activity against Tos–Gly–Pro–Arg–AMC substrate. The determined IC_50_ values for peptide **9** are independent of the substrate concentration (IC_50_ = 40 ± 4 µM at [S] = 50 µM and 40 ± 2 µM at [S] = 100 µM, respectively). The inhibition by peptide **9** is found to be in a reversible and noncompetitive way. Molecular modeling shows four cationic ammonium groups forming ion pairs and hydrogen bonds with negatively charged residues, such as Glu217, Asp60B, Asp147 and Glu217, respectively and completely block the central pore. Thus, it acts as inhibitor by preventing the substrate to reach the active sites in β-tryptase. Confocal laser scanning microscopy (CLSM) confirms that peptide **9** is mast-cell permeable and exhibits inhibitory activity within cells by suppressing their growth (Figure 8B).

### Heparin detection

**Figure 9:**
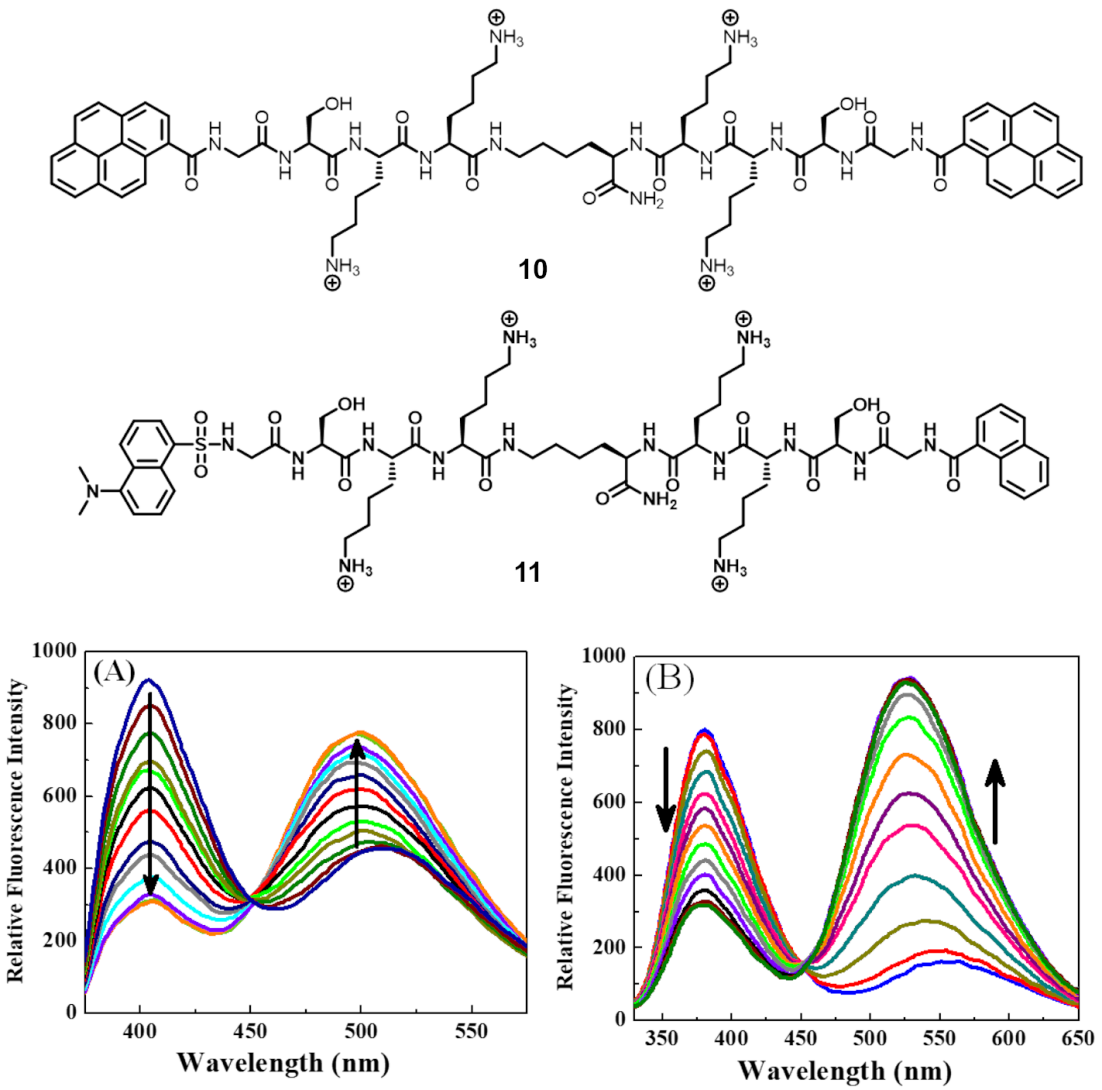
Top: Molecular structures of **10** and **11**; bottom: A) fluorescence emission spectra of **10** (1.0 µM, λ_ex_ = 340 nm); B) fluorescence emission spectra **11** (1.0 µM, λ_ex_ = 270 nm) with increasing concentration of heparin. Reproduced with permission from [56], Maity, D.; and Schmuck, C., “Fluorescent peptide beacons for the selective ratiometric detection of heparin”, Chem. – Eur. J. © 2016 Wiley‐VCH Verlag GmbH & Co. KGaA, Weinheim.

Heparin is a highly sulfated glycosaminoglycan with the highest negative charge density among known biomacromolecules. It is widely used as both a prophylactic and a therapeutic agent, especially as an anticoagulant in surgery [52–53]. But its overdose causes different adverse effects such as hemorrhages, thrombocytopenia, and hyperkalemia [54–55]. The Schmuck group reported lysine-rich peptide beacons for targeting the distinctive disaccharide unit of heparin in which one carboxylate and sulfate group are located on the same side [56]. The design of the peptide beacon consists of two Lys–Lys–Ser–Gly tetrapeptide arms attached through their C-terminus to a central lysine. Two heparin sensors **10** and **11** are developed by attaching the N-terminus of the peptide beacon with pyrenes and fluorescence resonance energy transfer (FRET) pair (naphthalene and dansyl), respectively for ratiometric detection of heparin (Figure 9). Sensors **10** and **11** switch from an open form to a folded form upon binding to heparin, which give rise to a ratiometric fluorescence signal. Upon the heparin binding, the emission switches from the pyrene monomer to the excimer for **10**, and the FRET process is enabled between a naphthalene donor and a dansyl acceptor for **11** (Figure 9A and Figure 9B). A minimum 30 nM of heparin can be detected by using **10** or **11**. The reported binding constants for **10** and **11** with heparin are 1.3 × 10^6^ M^−1^ and 2.9 × 10^6^ M^−1^, respectively. Sensors **10** and **11** are highly selective for the heparin detection over other biological analytes including such as adenosine, adenosine monophosphate (AMP), adenosine triphosphates (ATP), phosphate (Pi), pyrophosphate (PPi), ct-DNA, glucose, bovine serum albumin (BSA), glutamic acid and aspartic acid. They are also selective over the similar biological analytes hyaluronic acid or chondroitin sulphate. Sensores **10** and **11** even could detect heparin selectively from their mixtures and have been utilized for heparin detection in diluted bovine serum.

### Lipopolysaccharide detection

**Figure 10:**
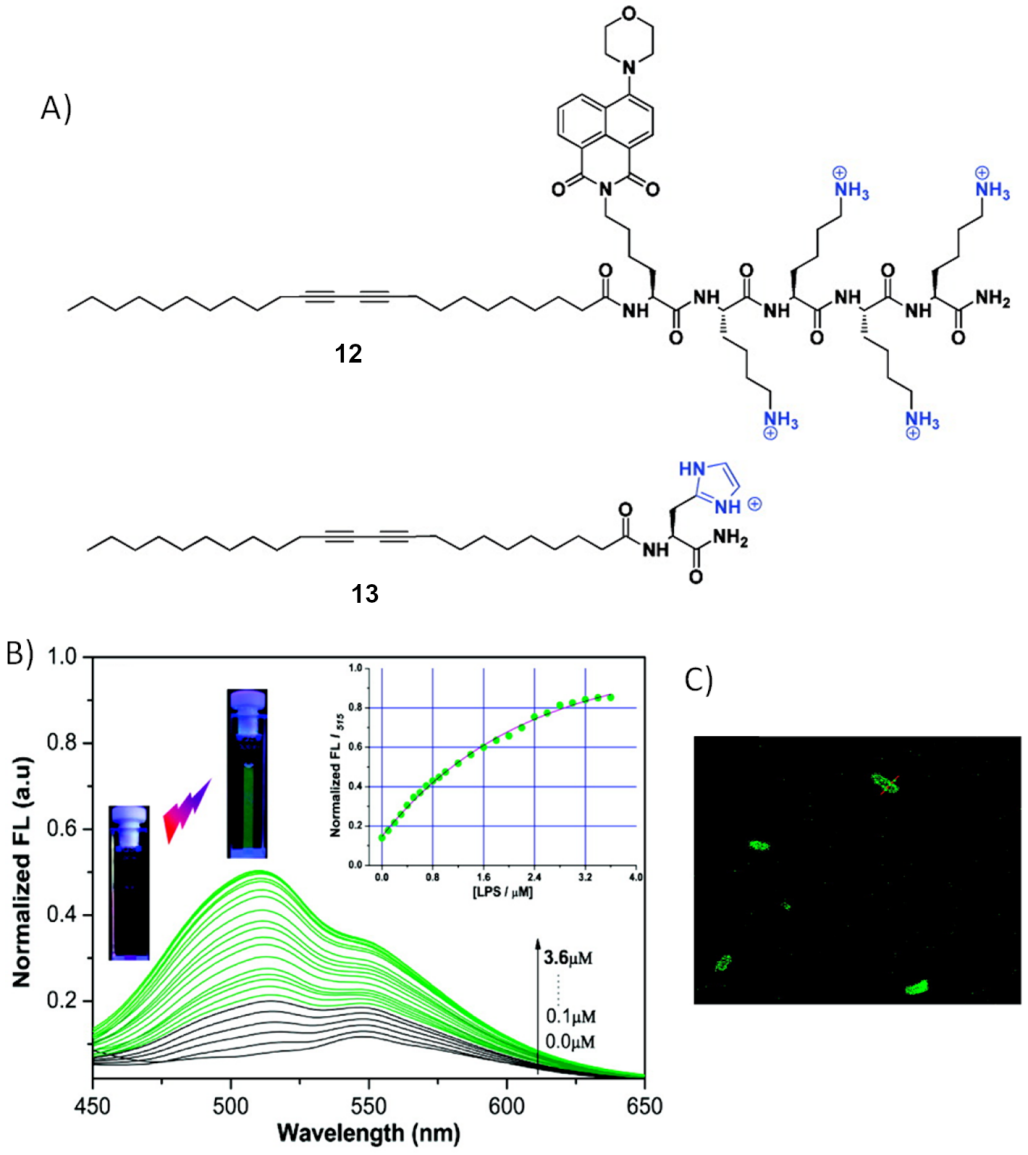
A) Structure of two peptide amphiphiles **12** and **13**; B) fluorescent spectra (λ_ex_ = 400 nm) from a titration of the PDA liposomes (molar ratio **12**/**13** = 1:9, total monomer concentration = 10.0 μM, 25 °C) in 10.0 mM DMSO/TBS (v/v = 1:4, pH 7.4). The insets show the normalized fluorescence intensity at 515 nm vs the concentration of LPS (0–3.6 μM) and the fluorescence turn-on; C) confocal luminescence image for *E. coli* DH5α after incubation with the PDA liposomes. Reprinted with permission from [59]. Copyright (2011) American Chemical Society.

Endotoxic lipopolysaccharide (LPS) is an important component in the outer cell membrane of Gram-negative bacteria [57]. It is a glycolipid consisting of a variable polysaccharide domain connected to a conserved glucosamine-based phospholipid called lipid A. It is highly negatively charged due to two phosphorylated groups in the lipid A part and carboxylated groups in the polysaccharide part. It is amphiphilic in nature as it contains minimum six fatty acid chains. It is toxic at higher concentration and has been identified in sepsis or septic shock in humans [58]. It induces a strong immune response in normal mammalian cells. Schmuck et al. reported cationic peptide-based polydiacetylene (PDA) liposomes for the turn on fluorescent detection of LPS in mixed solvent (DMSO/TBS buffer, 1:4) (Figure 10A) [59]. The liposomes are created by mixing two lipids in a ratio of 9:1. First lipid **12** is designed by attaching 10,12-tricosadiyonic acid to a penta-lysine oligopeptide bearing an aminonaphthalimide fluorophore on one lysine. The four cationic lysine residues are closely mimicking the structure of polymyxin B, which has a strong affinity to the negative LPS. The second lipid **13** is designed by connecting 10,12-tricosadiyonic acid to histidine. PDA liposomes are formed after UV-light-irradiated polymerization reaction between polydiacetylene. PDA liposomes turn colorless to red upon formation of cross-linked liposomes. The fluorescence of the naphthimide is completely quenched due to the energy transfer from the fluorophore to the cross-linked polymer. However, addition of LPS restores the fluorescence of the PDA liposomes to about 48% of the initial value (Figure 10B). The calculated binding constant from Stern–Volmer analysis of fluorescence titration is 1.5 × 10^6^ M^−1^. This system is very selective towards LPS as other analytes, including nucleotides, anionic sugars, and ctDNA did not increase fluorescent intensity except bovine serum albumin (BSA), which leads to a small enhancement. The size of the spherical self-assembled liposomes increases upon LPS binding, confirmed by DLS and AFM studies. Finally, PDA liposomes are utilized for fluorescence staining of the membrane of *E. coli* bacteria (Figure 10C).

### Lysosomes tracking

**Figure 11:**
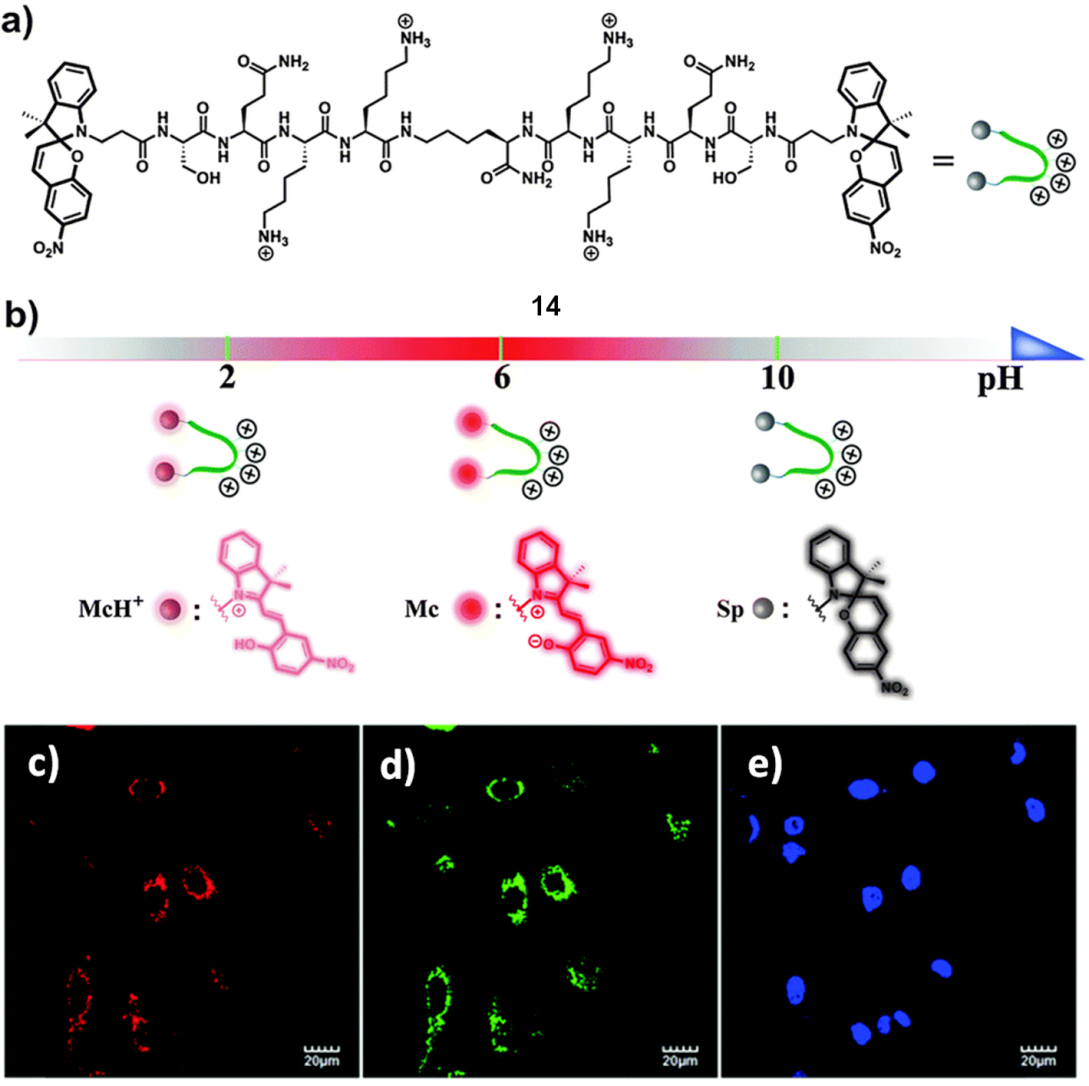
a) Molecular structure of peptide **14**; b) the coordinate represents the states of sensor at different pH values, purple sphere: protonated merocyanine (McH^+^), red sphere: normal merocyanine (Mc), and gray sphere: spiropyran at a ring-closed state (Sp); colocalization experiments using peptide **14**, LysoTracker Green DND-26 (LTG) and Hoechst 33258 in A549 cells. Cells were stained with c) 10 µM peptide **14** (channel 1: excitation: 515 nm, emission collected: 600–650 nm), d) 0.1 µM LTG (channel 2: excitation: 488 nm, emission collected: 500–550 nm) and e) 10 µg/mL Hoechst 33258 (channel 3: excitation: 405 nm, emission collected: 420–470 nm). Reproduced from [64], “A switchable peptide sensor for real-time lysosomal tracking”, © 2014 Chen et al., licensed under a Creative Commons Attribution 3.0 Unported Licence, https://creativecommons.org/licenses/by/3.0/. Published by The Royal Society of Chemistry.

Lysosomes participate multiple roles including intracellular transportation, metabolism, cell membrane recycling, and apoptosis [60]. Lysosomal lumen is acidic in nature and it is required for lysosomal enzyme activities. Deviation of lysosomal functions could result in diverse diseases, such as inflammation, tumor, silicosis, and various lysosomal storage diseases [61–63]. Therefore, closely monitoring/visualizing of the lysosomes acidic environment is required to study lysosome-related diseases. Spiropyran (SP) undergoes pH-induced interconversion among protonated nonfluorescent and colored ring-opened merocyanine (MCH^+^), fluorescent and colored ring-opened merocyanine (MC) and nonfluorescent and colorless ring-closed SP forms. Tian et al. in collaboration with the Schmuck group developed a bis-SP functionalized peptide **14** for pH monitoring in lysosomes and subsequent lysosomal imaging (Figure 11) [64]. Peptide **14** contains two symmetric lysine-rich arms attached via their C-terminus to a central lysine spacer. Under high acidic conditions (pH > 2), the peptide **14** shows a sharp absorption band at 410 nm and relatively weak fluorescence at 623 nm, corresponding to the MCH^+^ state. At pH 4–6, the fluorescence intensity increases by approximately 3-fold at 623 nm and absorption band red-shifts to 514 nm, which is characteristic of the MC form. At basic environment (pH > 10), absorption and fluorescence could not be observed, suggesting a complete conversion to the ring-closed SP state. The peptide **14** has been demonstrated pH responsive reversible red fluorescence staining of acidic lysosomes in A549 cell lines (Figure 11C).

## Conclusion

Schmuck group’s outstanding contributions in peptide-based fluorescent probes research field is summarized in this review. The development of peptide-based fluorescent probes has fascinated many research groups over the last two decades. Each group developed a different approach, which has led to an extensive set of fluorescent probes. Peptide-based probes based on FRET or environment-sensitive fluorophores provide a basis of reliable design strategies which are useful in number of applications including analyte detection, understanding molecular details of protein–peptide and protein–protein interactions. Recent developments of novel fluorophores and tailor-made receptors have opened the possibility of designing peptide-based new chemical tools for biological analysis and clinical diagnosis, and it is exciting to contemplate what will become possible in the next decade.
